# NF-Y loss triggers p53 stabilization and apoptosis in HPV18-positive cells by affecting E6 transcription

**DOI:** 10.18632/oncotarget.9974

**Published:** 2016-06-13

**Authors:** Paolo Benatti, Valentina Basile, Diletta Dolfini, Silvia Belluti, Margherita Tomei, Carol Imbriano

**Affiliations:** ^1^ Dipartimento di Scienze della Vita, Università di Modena e Reggio Emilia, 41125 Modena, Italy; ^2^ Dipartimento di Bioscienze, Università degli Studi di Milano, 20133 Milano, Italy

**Keywords:** NF-Y, CCAAT-box, HPV18, p53, gene transcription

## Abstract

The expression of the high risk HPV18 E6 and E7 oncogenic proteins induces the transformation of epithelial cells, through the disruption of p53 and Rb function. The binding of cellular transcription factors to *cis*-regulatory elements in the viral Upstream Regulatory Region (URR) stimulates E6/E7 transcription. Here, we demonstrate that the CCAAT-transcription factor NF-Y binds to a non-canonical motif within the URR and activates viral gene expression. In addition, NF-Y indirectly up-regulates HPV18 transcription through the transactivation of multiple cellular transcription factors. NF-YA depletion inhibits the expression of E6 and E7 genes and re-establishes functional p53. The activation of p53 target genes in turn leads to apoptotic cell death. Finally, we show that NF-YA loss sensitizes HPV18-positive cells toward the DNA damaging agent Doxorubicin, *via* p53-mediated transcriptional response.

## INTRODUCTION

The infection with high-risk human papillomavirus (HPV), usually of type 16 and 18, is the main cause for cervical cancer [1, 2]. In the course of cancer development, the HPV genome is frequently integrated into host-cell DNA. The open reading frame (ORF) of E2, E4, E5, and part of L2 genes are deleted after integration. Differently, the E6 and E7 early genes are preserved and encode for oncoproteins, which are responsible for initiation and progression of cervical cancer [2, 3]. E6 and E7 can independently immortalize human cells, but their cooperation results in a robust increase in transforming activity in most types of primary cells [4, 5]. E7 interacts with and degrades retinoblastoma (Rb), thus triggering E2F-dependent gene transcription of S-phase genes [6, 7]. Prominent functions of E6 are the degradation of the oncosuppressor p53 [8] and the inhibition of its transcriptional activity [9]. In addition, E6 degrades the pro-apoptotic protein BAX, leads to transcriptional activation of hTERT [10] and inhibits the degradation of SRC-family kinases [11].

The expression of E6 and E7 is transcriptionally controlled by specific elements within the viral Long Control Region (LCR), also termed Upstream Regulatory Region (URR), a non-coding sequence between the ORFs of L1 and E6 genes. To date, only a handful of Transcription Factors (TFs) have been associated to positive regulation of HPV transcription, such as AP1, SP1, Oct1, YY1 and NF1 [12]. The TRANSFAC database revealed that other TFs could control HPV early gene expression, and ChIP assays detected FOXA1 and MYC binding to HPV18 URR [13]. More recently, ChIP-seq data from Hela-S3 cells have been re-analyzed to determine the occupancy of TFs on the integrated HPV18 genome [14]. Elk1, IRF3, MafK, MAZ, USF2 and ZKSCAN have been pointed out as possible HPV18 regulators.

The CCAAT sequence-specific transcription factor NF-Y activates the expression of multiple genes involved in cell proliferation [15]. NF-Y is composed of three subunits, NF-YA, NF-YB and NF-YC, whose association is necessary for DNA binding and transcriptional activity. A connection between NF-Y and p53 exists [16]. First, the inactivation of NF-YA, the regulatory subunit of the complex, triggers DNA damage and the activation of p53-mediated apoptosis [17]. Second, NF-Y and p53 interaction is required for the repression of cell cycle CCAAT-genes following DNA damage (18–21). Finally, NF-YA is the target of PANDA, a p53-induced lncRNA involved in the regulation of apoptosis and senescence [22, 23].

Distinct effects were observed on cell cycle and gene transcription following NF-YA or NF-YB loss in wtp53-positive cells [17]. NF-YA knock-down suppresses cell growth by impairing S phase progression [17, 24]. Replication defects are coupled with DNA damage response, p53 activation and apoptotic cell death. Differently, NF-YB inactivation triggers a delay in the G2/M progression without p53 activation, DNA damage response or overt apoptosis. Nevertheless, the GO term “*apoptotic program*” is significanly enriched in genes up-regulated both by NF-YA and NF-YB knock-down [17].

Here, we show a new connection between NF-Y and p53 in HPV18+ cancer cells. NF-Y binds viral URR and transcriptionally induces HPV18 genes. By mutational analysis and Chromatin Immunoprecipitations (ChIPs), we identified a non-consensus NF-Y binding motif within the URR. NF-YA loss reduces the expression of E6 and E7 viral genes and results in the re-activation of a functional p53. This in turn triggers apoptotic cell death. Analysis of gene expression profiles in NF-YA-inactivated Hela cells indicates that NF-Y transactivates other key TFs driving the expression of viral genes. Finally, we show that NF-YA loss sensitizes Hela cells to Doxorubicin treatment.

## RESULTS

### NF-YA loss induces p53 and apoptotic cell death in Hela cells

**Figure 1:**
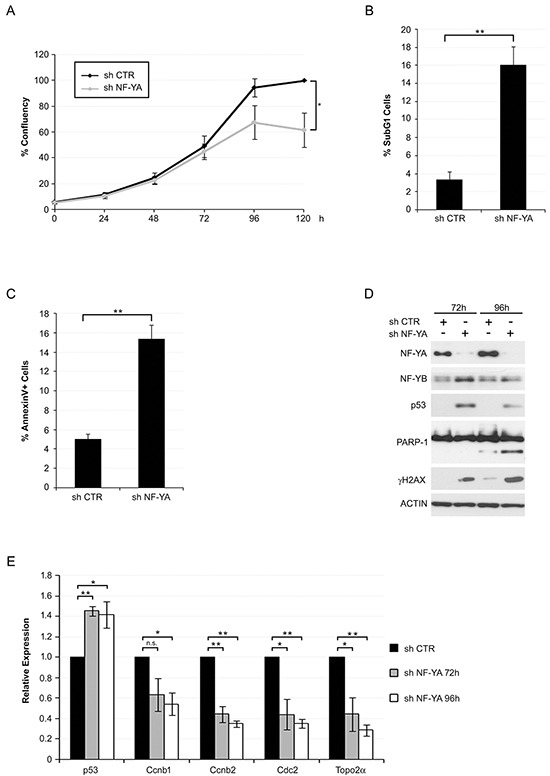
NF-YA inactivation in Hela cells triggers activation of apoptotic cell death and p53 **A.** Growing curve of Hela cells infected with shCTR and shNF-YA. Time points are indicated. Statistical significance was calculated with independent t-test at 120h (* p < 0.05). **B.** Percentage of subG1 events determined by Propidium Iodide-FACS analysis of Hela cells 96h post infection with shCTR and shNF-YA lentiviral particles. Statistical significance was calculated with independent t-test (** p < 0.01). **C.** Percentage of AnnexinV-positive cells 96h post infection with shCTR and shNF-YA. Statistical significance was calculated with independent t-test (** p < 0.01). **D.** Expression levels of the indicated proteins in Hela whole cell extracts 72h and 96h post-infection with shCTR and shNF-YA. Actin was used as loading control. **E.** q-RT PCR analysis of the indicated transcripts 72h and 96h post-infection with shCTR and shNF-YA. The housekeeping hRpl19 gene has been used for normalization. Statistical significance is calculated with independent t-test (* p < 0.05; ** p < 0.01). Error bars indicate Standard Error of the Mean (SEM).

We previously showed that p53-lacking HCT116 cells were less sensitive to apoptosis following NF-YA inactivation, compared to isogenic wt p53 cells [17]. In the course of that study, we noticed that NF-YA inactivation in Hela cells, which do not express p53 protein, led to a strong decrease in cell proliferation. Here we observed that SubG1 events raised from about 3% in cells infected with scramble shRNA (SHC) to 16% upon NF-YA loss (shNF-YA) (Figures 1A and 1B). Cytofluorimetric analysis of AnnexinV staining confirmed that about 16% of shNF-YA cells were apoptotic, as observed in p53+ cells [17] (Figure 1C). Western Blot analysis of PARP1, whose cleavage is a hallmark of caspase-mediated apoptosis, further confirmed the activation of apoptosis (Figure 1D). We reasoned that NF-YA loss could re-activate p53, whose gene status is wt in Hela cells. Indeed, the expression of γH2AX, marker of DNA damage response, and p53 increased in shNF-YA cells compared to control cells (Figure 1D). In order to rule out the possibility that off-target effects were causing the described effects, we used pooled shRNAs targeting different exons of NF-YA. p53 and apoptosis raised also in these experimental conditions (Supplementary Figure S1A). qRT-PCRs were performed to assess mRNA levels of p53. In shNF-YA cells, p53 transcription significantly increased, in opposition to NF-Y-regulated cell cycle genes (Ccnb1, Ccnb2, Cdc2 and Top2A) (Figure 1E). Similarly, NF-YA inactivation resulted in activation of apoptosis and p53 re-expression in C4-I cell line, derived from a HPV18+ squamous cell carcinoma of the uterine cervix (Supplementary Figure S1B).

**Figure 2:**
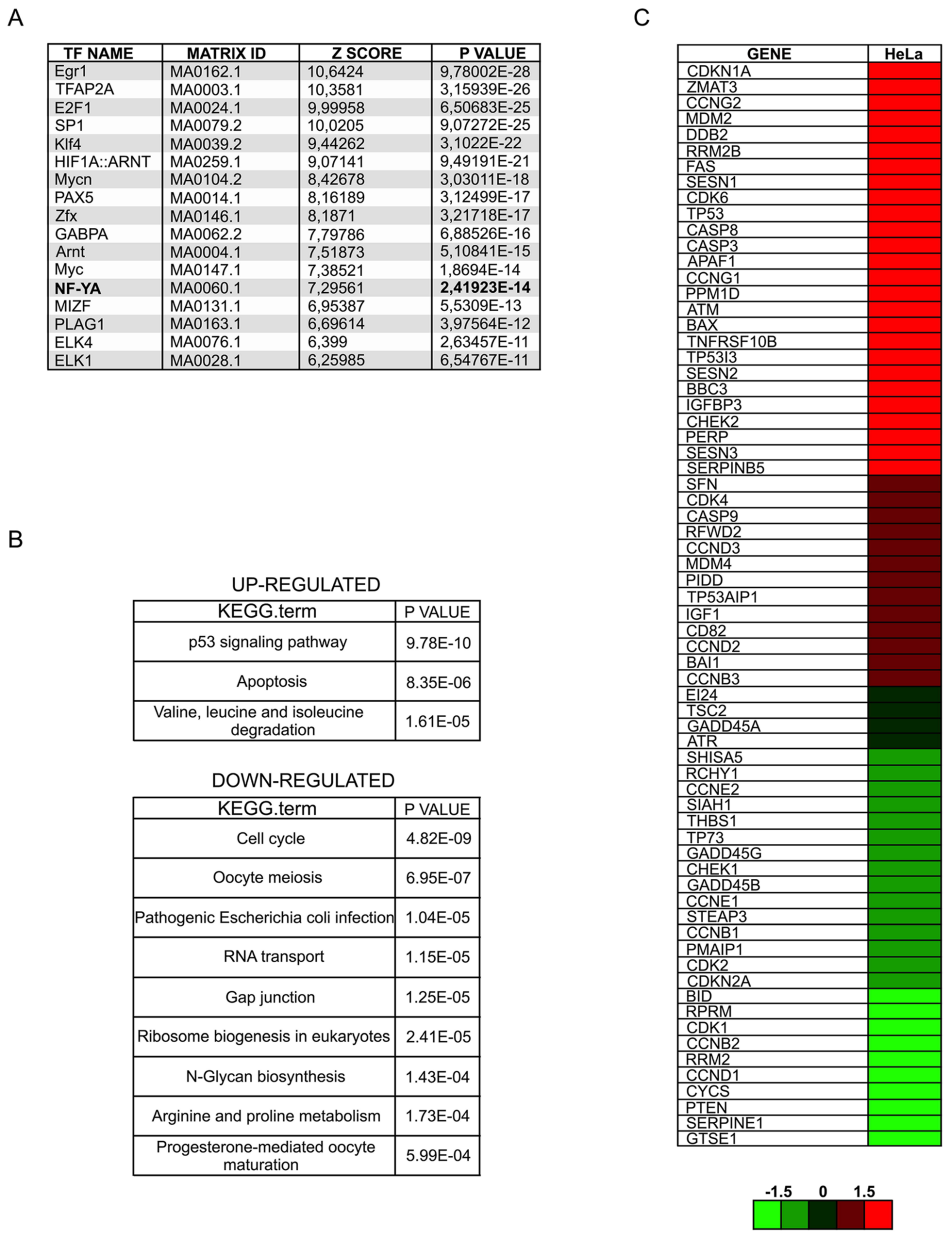
NF-YA loss activates a p53-dependent transcriptional response **A.** *Pscan* analysis of Transcription Factors Binding Sites (TFBS) with relative p-values in down-regulated genes following NF-YA inactivation by shRNA. **B.** KEGG analysis of up- and down-regulated genes retrieved from gene expression profiles of NF-YA-inactivated cells. **C.** Heat map of p53-target genes upon NF-YA abrogation.

**Figure 3:**
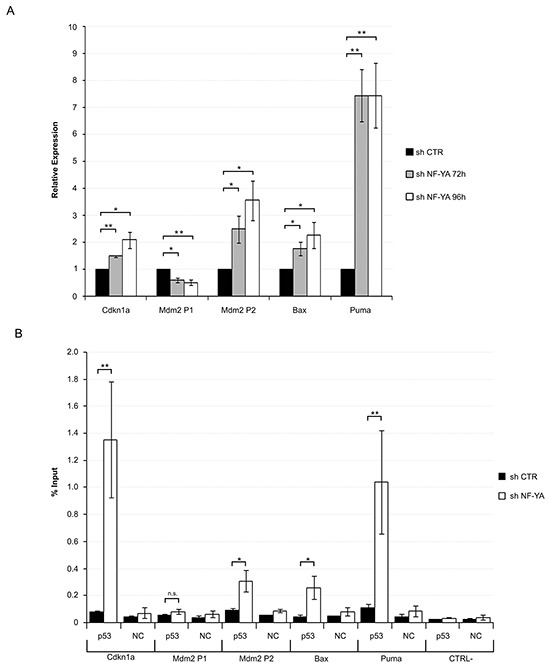
Activation of functionally active p53 in NF-YA-inactivated Hela cells **A.** qRT-PCR analysis of *bona fide* p53 target genes 72h and 96h post-infection with shCTR and shNF-YA. Amplified genes are indicated. Statistical significance was calculated with independent t-test (* p < 0.05; ** p < 0.01). **B.** ChIP analysis of p53 binding to the regulatory regions of Cdkn1a, Mdm2-P1, Mdm2-P2, Bax and Puma genes in shCTR and shNF-YA cells 96h after infection. CTRL- represents a CCAAT-less negative control region, localized at about 5000bp upstream of the Myc gene. The p53 enrichment was determined as percentage of IP recovery. Statistical significance was calculated with independent t-test (* p < 0.05; ** p < 0.01). Error bars indicate SEM.

A functional p53 would be expected to drive expression of its own target genes: we therefore analyzed Affymetrix gene expression profiles, obtained after 72 hours from infections with scramble and NF-YA-targeting shRNAs [25]. 1492 genes were down-regulated and 1500 genes were up-regulated upon NF-YA loss, considering a threshold of 1.3-fold difference and a p-value smaller than 0.1. We analyzed the affected promoters by *pscan,* a software for the identification of enriched TFBS (Transcription Factors Binding Sites) [26]. NF-Y sites were over-represented in down-regulated genes (p-value= 2,41923E-14), indicating that NF-Y removal significantly decreased CCAAT-driven transcription (Figure 2A). Additional TFBS were found, hinting that NF-YA loss could inhibit the expression of genes regulated by other TFs. In agreement with this, we know that: i) NF-Y transcriptionally activates specific TFs, such as E2F1 and Myc, and ii) a transcriptional partnership exists between NF-Y and other growth-controlling TFs [24, 25, 27-29]. KEGG analysis was performed with the three sets of up and down-regulated genes, and *cell cycle* was the major term identified in this cluster (Figure 2B), as already observed in HCT116 cells [17]. The NF-Y motif was less represented in activated genes (p-value= 9,96824E-05), suggesting that increased gene expression was at least in part due to indirect effects. Importantly, the terms *p53 signaling pathway* and *apoptosis* were identified as the major represented KEGG terms in shNF-YA cells. These data support the hypothesis that NF-YA abrogation triggers the activation of functional p53. The heat map in Figure 2C highlights the differential expression of p53-target genes upon NF-YA loss. These results were validated by qRT-PCRs on *bona fide* p53-targets. The levels of Cdkn1a (p21^Waf1/Cip1^), Bax, Puma and the p53-dependent inducible Mdm2-P2, but not the p53-independent constitutive Mdm2-P1 transcript [30], significantly increased (Figure 3A). To verify whether p53 was functionally active, its association to regulatory regions of target genes was investigated by ChIP. A robust increase in p53 binding to the promoters of Cdkn1a, Mdm2-P2, Bax and Puma was induced by NF-YA depletion (Figure 3B).

Taken together, these results indicate that NF-YA inactivation in HPV18+ cells reactivates a functional p53, which in turn induces the expression of anti-proliferative and pro-apoptotic genes.

### NF-Y regulates the transcription of HPV oncogenic genes

**Figure 4:**
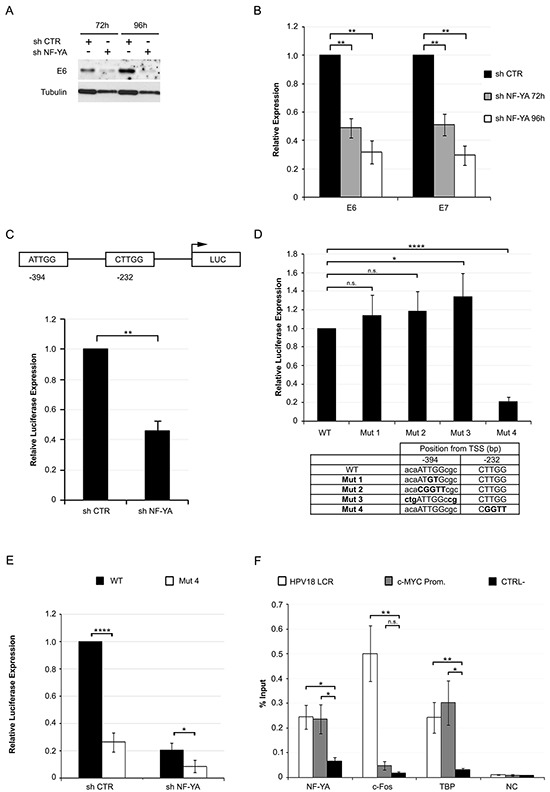
NF-Y transcriptionally controls the expression of HPV18-URR driven genes **A.** Western Blot analysis of E6 protein in whole cell extracts from Hela infected with shCTR and shNF-YA for 72h and 96h. Tubulin was used as loading control. **B.** Relative expression levels of E6/E7 genes normalized to the hRpl19 transcript in shNF-YA cells versus shCTR, arbitrarily set at 1. Statistical significance was determined with independent t-test (** p < 0.01). **C.** Upper panel: schematic representation of CCAAT boxes position in HPV18-URR, cloned upstream of the luciferase (LUC) reporter gene. Lower panel: relative HPV18-URR-driven luciferase activity in shCTR and shNF-YA cells. Statistical significance was calculated with independent t-test (** p < 0.01). **D.** Relative luciferase expression of mutant promoters with respect to wt HPV18 promoter. Statistical significance was calculated with independent t-test (* p < 0.05; **** p < 0.0001). The table indicates the position and sequence of the two wt and mutated NF-Y-motives. **E.** Relative luciferase activity of wt and mut4 HPV18 URR in shCTR and shNF-YA cells. **F.** ChIP analysis of NF-YA, c-FOS and TBP binding to HPV18-LCR, c-Myc promoter and negative control region (CTRL-) in Hela cells. Enrichment was calculated as percentage of IP recovery. Statistical significance was calculated with independent t-test between promoters of interest and CTRL- region (* p < 0.05; ** p < 0.01). Error bars indicate SEM.

Altered regulation of the E6 gene could be the cause of p53 re-activation in NF-YA depleted cells. Western blot and qRT-PCR analysis showed a time-dependent decrease in E6 levels following NF-YA inactivation in Hela and C4-1 cells (Figure 4A, 4B and Supplementary Figure S1C, S1F). We detected a similar decrease in E7 mRNA expression, which is also controlled by the URR.

Genomic analysis identified two putative NF-Y binding sites within the URR: the first, at −394bp from the TSS, is an inverted CCAAT (ATTGG) sequence, conserved in both African (Af) and non-African (non-Af) HPV18 lineages [31] The second one, at −232bp, is represented by a canonical ATTGG motif in the Af and non-canonical CTTGG sequence in the non-Af lineage (Supplementary Figure S2). To assess gene expression driven by URR, we used the HPV18-URR pGL3-Luciferase reporter plasmid, which contains the upstream ATTGG and the downstream CTTGG sequences [32]. NF-YA inactivation significantly reduced HPV18-URR-Luc activity, with respect to control cells (Figure 4C). Thereafter, we mutated the −394 element either in the core ATTGG -to ATGTG (mut1) or CGGTT (mut2)- or in the flanking nucleotides on both the 5′ and 3′ ends (mut3), potentially improving the quality of the putative binding site [33]. We also mutated the −232bp element from CTTGG to CGGTT (mut4). These constructs were transfected in Hela cells: reporter activity of mut1 or mut2 was not reduced, and mutations of the flanking regions marginally enhanced HPV18 activity. Differently, the activity of mut4 was substantially reduced (Figure 4D). NF-YA loss decreased mut4-Luc activity (Figure 4E), hinting at NF-Y indirect mechanisms occurring in URR regulation.

Having established the functionality of a CCAAT-like DNA element, we wished to ascertain whether the role of NF-Y on HPV18 transcription was direct. Analysis of Hela-S3 ENCODE ChIP-Seq data scored negative in the HPV18 genome area, either for NF-YA or NF-YB [14]. Nevertheless, we decided to perform qChIPs in Hela cells with anti-NF-YA antibody (Figure 4F). A significant enrichment in NF-YA binding to HPV18-LCR was observed over control IgG, similar to the levels found in the human Myc CCAAT-promoter bound by NF-Y [24]. As positive controls, the same viral region showed binding of FOS and TBP, known to associate to HPV18-LCR [14]. All together, these results suggest that NF-Y directly affects HPV18 transcription by binding to a non-canonical CCAAT element within the URR region.

### NF-YA inactivation affects the expression of TFs involved in HPV18 transcription

**Figure 5:**
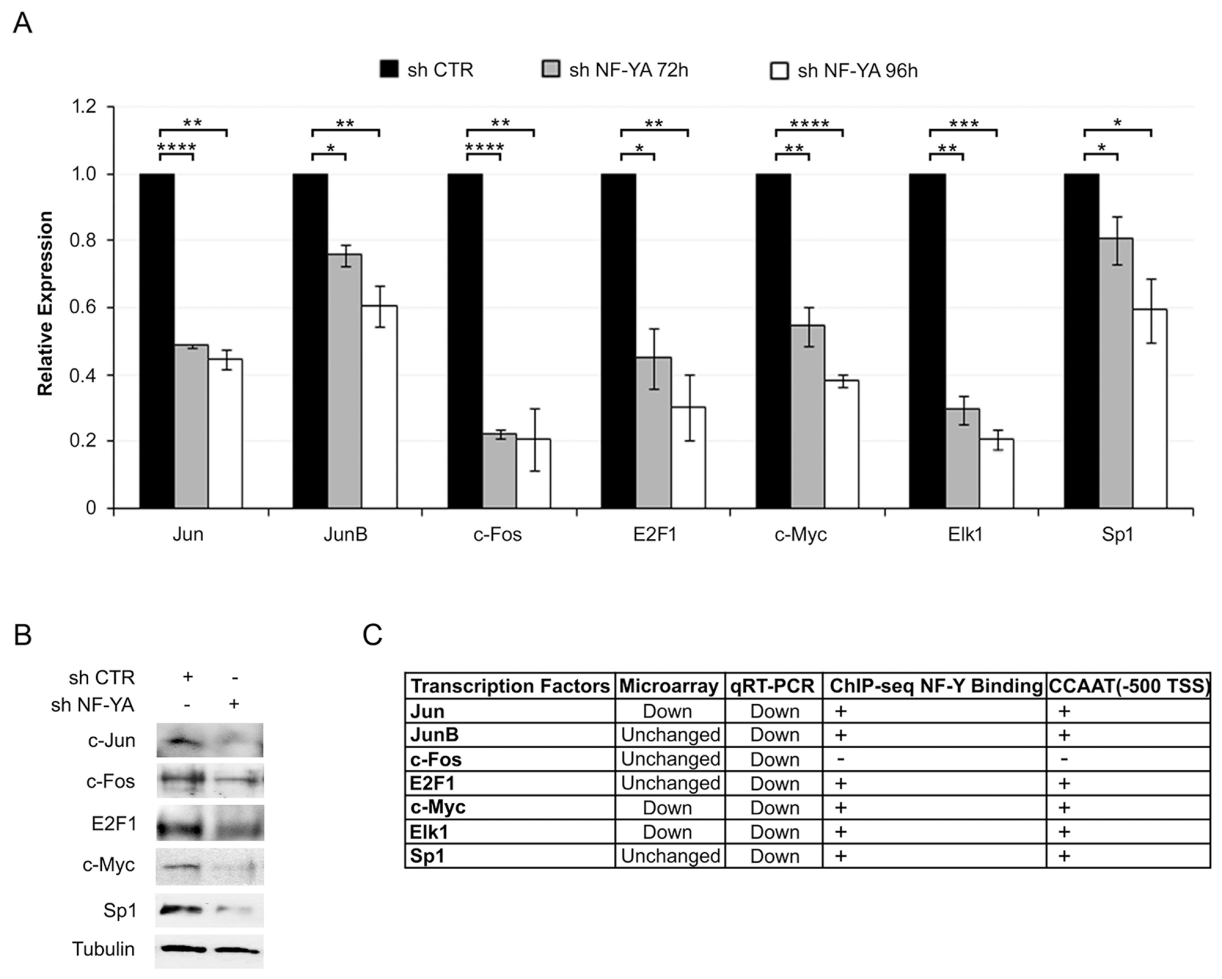
NF-YA inactivation affects the expression of key transcription factors regulating HPV18-URR activity **A.** qRT-PCR analysis of the indicated genes 72h and 96h post-infection with shCTR and shNF-YA. hRpl19 has been used as reference gene. Statistical significance was calculated with independent t-test (* p < 0.05; ** p < 0.01; *** p < 0.001; **** p < 0.0001). Error bars indicate SEM. **B.** Western blot analysis of the indicated proteins in shCTR and shNF-YA cells 96h post-infection. **C.** The table indicates the effect of NF-YA loss on gene transcription of the indicated transcription factor (TF), observed by qRT-PCR and gene expression profiling, the binding of NF-Y (ENCODE ChIP-seq data) and the presence (+) or not (−) of canonical NF-Y binding site in TF-promoters (−500bp from the TSS).

We next wondered whether NF-Y could be involved in the regulation of other TFs identified as regulators of viral genes. AP1 (Jun/Fos), E2F1, SP1, Myc and Elk1 are associated to HPV18-LCR by ChIP-seq analysis [14], and some of them are indispensable for viral gene expression [12, 34, 35]. Jun, JunB and Fos, members of the AP1 complex, E2F1, Myc, Elk1 and SP1 were indeed down-regulated at the transcriptional level following NF-YA inactivation in Hela cells (Figure 5A). Western blot analysis showed a decrease in protein levels as well (Figure 5B). With the exception of Fos, all the other TFs have canonical NF-Y-motives within their regulatory regions. Consequently, we checked whether NF-Y could function as direct transcriptional regulator. ENCODE data from Hela-S3 ChIP-seq are positive for NF-Y binding in all of the analyzed genes, Fos excluded (Figure 5C). Thus, in addition to a direct role, NF-Y could indirectly induce HPV18 transcription through the transactivation of TFs that cooperate in viral transcription.

### NF-YA loss sensitizes cells to Doxorubicin-induced cell death

**Figure 6:**
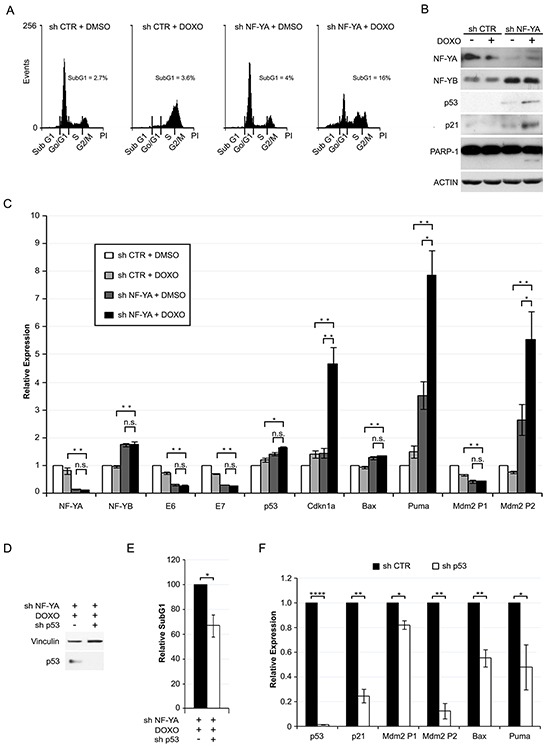
NF-YA loss sensitizes Hela cells to Doxorubicin-induced p53-dependent cell death **A.** DNA distribution analysis of Propidium Iodide-stained Hela cells infected with shCTR and shNF-YA for 72h and then treated with DMSO or 0.1 μM Doxorubicin (DOXO). The percentages of SubG1 events are indicated. Shown images are representative of three independent experiments. **B.** Western blot analysis of whole cell extracts in the experimental conditions described above. Antibodies are indicated. Actin was used as loading control. **C.** qRT-PCR relative expression analysis of p53 target genes in shCTR and shNF-YA cells treated or not with DOXO. The housekeeping hRpl19 gene has been used for normalization. The expression levels of control cells (shCTR +DMSO) have been arbitrarily set at 1. **D.** p53 expression levels in NF-YA-inactivated cells infected with shCTR and shp53 and treated with DOXO. **E.** Effects of p53 loss (shp53) on SubG1 events in NF-YA-inactivated cells treated with DOXO. The percentage of SubG1 in NF-YA-inactivated cells infected with shCTR has been arbitrarily set at 100%. **F.** qRT-PCR analysis of the indicated transcripts in NF-YA/p53 double knocked down cells versus NF-YA-inactivated cells (set at 1), following DOXO treatment. Statistical significance was calculated with independent t-test (* p < 0.05; ** p < 0.01; **** p < 0.0001;). Error bars indicate SEM.

We then investigated whether p53 activation *via* shNF-YA could sensitize HPV18+ cells toward DNA-damaging agents. Hela cells were insensitive to 0.1μM Doxorubicin (Doxo), a DNA intercalator used in clinics for a broad spectrum of tumors. Indeed, p53 expression and apoptosis did not increase in Doxo versus control cells (Supplementary Figure S3). Untreated and Doxo-treated cells were infected with shRNA lentiviral particles at low MOI, in order to lower NF-YA expression without inducing strong apoptotic cell death (subG1~4% in shNF-YA untreated cells) (Figure 6A). The concurrent treatment with Doxo and shNF-YA (shNF-YA+Doxo cells) activated an evident cell death response (subG1~16%). Consistently, the expression levels of p53, p21 and cleaved-PARP1 increased in shNF-YA+Doxo cells (Figure 6B). qRT-PCRs showed a significant increase in the levels of p53-target genes in shNF-YA+Doxo cells, compared to both single treatments (shCTR+Doxo and shNF-YA+DMSO) (Figure 6C). Differently, no synergistic effect was observed on the transcriptional activation of Bax. The expression of NF-YA, E6 and E7 genes decreased following NF-YA loss, as expected, and did not change upon Doxo administration. p53 knock-down in NF-YA-inactivated cells significantly reduced SubG1 events triggered by Doxo treatment (Figure 6D, 6E). Consistently with p53 loss, the transcription levels of p53-target genes decreased (Figure 6F).

In summary, even incomplete ablation of NF-YA leads to increased sensitivity to a DNA-damaging agent, *via* activation of a p53-mediated transcriptional response.

## DISCUSSION

Our study shows that the abrogation of NF-YA triggers p53-mediated apoptosis in HPV18+ cells. We demonstrate that NF-Y is a transcriptional activator of HPV18-URR gene expression by binding to a non-canonical inverted CCAAT box, located at −232bp from the TSS. Interestingly, this NF-Y-bound sequence (CTTGG) partially overlaps with a previously identified NF1 binding site (TTGGCT) (Supplementary Figure S4). Coherently with our results, mutation of the NF1 site, that abolishes NF1 binding but preserves the NF-Y motif (CTTGGta), does not reduce the activity of HPV18 URR in Hela cells [36]. This hints at a predominant role of NF-Y within these two overlapping TFBS.

HPV high risk types 16 and 18 share some common transcriptional regulators, such as SP1 and AP1 [12]. Nevertheless, neither canonical NF-Y motives nor the non-conserved element here described were identified in HPV16 URR (Supplementary Figure S4).

The restoration of active p53 can be achieved in HPV18+ cells through pharmacological treatments, such as Celecoxib or the combination of Actinomycin D with Leptomycin B [37, 38]. Also the targeting of TFs involved in HPV transcriptional regulation, such as NF90/NF45 [39, 40], has been used as p53-activating strategy. We established here that NF-Y targeting reactivates functional p53 as well. Note that NF-Y inactivation induces p53 post-translational modifications [17, 41] and reduces the basal expression of CCAAT box-containing proteasome genes [42]. Therefore, it is likely that these mechanisms can cooperate with E6 down-regulation to restore functional p53 upon NF-YA loss.

We also investigated the effects of NF-YA abrogation on other p53 family members, p63 and p73. The level of p63 mRNA, already low in Hela control cells, was further reduced following NF-YA loss. At the protein level, we observed a decrease in ΔNp63, the only isoform we detected by Western blot in control cells (Supplementary Figure S5) [43, 44]. This result is consistent with the established role of NF-Y as transcriptional activator of the ΔNp63 promoter [45]. Also p73 mRNA levels were lowered by shNF-YA infection, presumably as a consequence of reduced expression of E2F1, known p73 transcriptional activator [46] (Supplementary Figure S5).

NF-Y plays an important role in the transcriptional control of genes encoded by the genome of different viruses, such as the human Herpes Simplex Virus type 1 (HSV-1) [47], the Epstein Barr Virus (EBV) [48], the Kaposi's sarcoma-associated herpesvirus (KSHV) [49] and the Minute Virus of Mice (MVM) [50]. The transcriptional control of the MVM P4 promoter is determined by the association of NF-Y to an unusual site -CCAAC-, similarly to what we described here. The atypical NF-Y motif found in the non-Af lineage is functional, presumably thanks to nucleotides on both the 5′ and 3′ flanking sides. Since a canonical inverted CCAAT box is present in Af, it is likely that the −232bp element has preserved its transcriptional function in both lineages. In general, NF-Y requires a perfect match of the pentanucleotide, as well as flanking sequences, for efficient DNA-binding [51]. However, the 3D structure of the NF-Y complex bound to CCAAT indicates that the final T is the only nucleotide of CCAAT not contacted in a sequence-specific way by NF-YA [52]. This suggests that a higher degree of tolerance is allowed at this position. Moreover, ChIP-Seq data clearly recovered a vast majority of CCAAT-containing locations (>80%), but non canonical sites usually have a different nucleotide instead of the final T [25].

The inhibition of the interactions of NF-Y to its binding site through DNA sequence-specific conjugated polyamides is a successful strategy to affect the transcription of specific CCAAT-promoters [53–55]. It is therefore tempting to speculate that drug specific inhibition of NF-Y association to its binding site in HPV18-URR could represent and interesting therapeutic strategy against HPV+ cancer cells.

**Figure 7:**
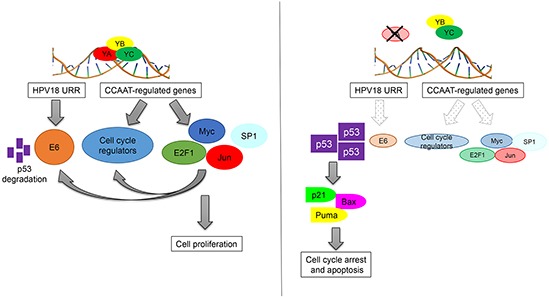
Schematic representation of direct and indirect functions of NF-Y in the control of HPV18+ cells proliferation.

In summary, our results identify an additional mechanism through which NF-Y and p53 are connected in HPV+ cancer cells (Figure 7). NF-YA inactivation might represent an interesting anti-tumor strategy to induce apoptosis in high-risk HPV infected cancer cells, or to sensitize them to conventional anti-tumor drugs, through the re-establishment of p53-mediated cell death.

## MATERIALS AND METHODS

### Cell culture, treatments and shRNA inactivation

The cervical cancer Hela and C4-I HPV18+ cell lines were maintained in complete Dulbecco's Modified Eagle's Medium (DMEM) with 10% Foetal Bovine Serum (FBS) and grown at 37°C in a humidified incubator containing 5% CO2 [56]. Doxorubicin (Sigma Aldrich) was solved in DMSO and added to the cells at the indicated concentrations for 24 hours.

Hela and C4-I cells were infected with PLKO1 shRNA NF-YA (targeting exon 6) lentiviral particles (MOI=8), as previously described [17, 25]. A combination of two different PLKO1 shRNAs (Sigma Aldrich) targeting exons 8 and 10 of NF-YA was used (MOI=6 for each shRNA) (Supplementary Figure S1A). p53 inactivation was achieved by infecting cells with PLKO1 p53-targeting shRNA (Addgene, #19119) (MOI=8). The puromycin resistance cassette was replaced with the EGFP cassette, as previously described [17].

### Cell proliferation analysis

10.000 cells have been seeded into 24-well plates and infected shCTR and shNF-YA lentiviral particles. At the indicated time points, cells have been fixed with Crystal Violet solution (0.25% Crystal Violet, 20% methanol in water) for 1h and then washed 6 times with water. Cell layers have been than resuspended in 1ml of acid isopropanol (HCl 0.1M, 20% isoprophanol) and the adsorbed Crystal Violet has been quantified with spectrophotometer at a λ of 540 nm.

### Flow cytometry

For the determination of cell cycle progression, cells were stained with Propidium Iodide (PI), as previously described [57]. Apoptotic cells were detected by FACS using Annexin V-PE conjugate (BD Biosciences, Becton Dickinson Italia, Milan, Italy), following the protocol of the manufacturer.

### Immunoblotting

For whole cell lysates, cells were resuspended in 1X SDS sample buffer (25mM Tris–HCl pH 6.8, 1.5mM EDTA, 20% glycerol, 2% SDS, 5% b-mercaptoethanol, 0.0025% Bromophenol blue). For Western blot analysis, equal quantity of cell lysates were separated by SDS-polyacrylamide gel electrophoresis, transferred to PVDF membrane (VWR) and probed with the following primary antibodies: anti-NF-YA (Santa Cruz, sc-17753), anti-NF-YB (GeneSpin), anti-p53 (Santa Cruz, sc-126), anti-PARP1 (Santa Cruz, sc-8007), anti-H2AX (Santa Cruz, sc-101696), anti-p21 (Millipore, 05-345), anti-E2F1 (Bethyl, A300-766A), anti-cJun (Bethyl, A302-958A), anti-cMyc (Santa Cruz, sc-764), anti-Fos (Santa Cruz, sc-52), anti-p63 4A4 (Santa Cruz, sc-A0311), anti-actin (Santa Cruz, sc-1616), anti-tubulin (Sigma Aldrich, T-6074), anti-E6 (Santa Cruz, sc-365089). Chemiluminescent detection reagent was purchased from Millipore Spa (Luminata Classico and Forte Western HRP).

### Chromatin immunoprecipitation (ChIP)

ChIPs were performed as previously described [17]. 4 μg of the following antibodies were added to each IP and incubated overnight at 4°C: anti-NF-YA (Santa Cruz, sc-10779), anti-p53 (Santa Cruz, sc-126) and anti-IgG (Santa Cruz, sc-2027), used as control for non-specific interactions. Immunoprecipitated DNA was resuspended in TE buffer, and *Real Time* PCR analyses were performed with the following primers:

**Table:** 

Promoter		5′-3′ Sequence	bp lenght	Tm
Cdkn1a	For	ATTCCCCTAC CCCATGCT	153	60
	Rev	GCCAGAAAG CCAATCAGAG		
Mdm2 P1	For	CAGCCAAACC CAAACATTCT	184	56
	Rev	CGCTGGAGT TGTACCCAAAT		
Mdm2 P2	For	CAGGTAAGC ACCGACTTGCT	190	56
	Rev	GCTGGAATCT GTGAGGTGGT		
Bax	For	CCCCCGTCACT TTATCTGCT	103	56
	Rev	GGGTTCTAGGGG ATCAGGAG		
Puma	For	TCAGTGTGTGTG TCCGACTGTC	96	60
	Rev	GGCAGGGC CTAGCCCA		
HPV18 LCR	For	CTCTTTGGC GCATACAAGG	90	60
	Rev	GGGAGTGGA TATAGTTGTGCAA		
c-Myc	For	TATCTACACTAACAT CCCACGCTCTG	192	60
	Rev	CATCCTTGTCCTGT GAGTATAAATCATCG		
CTRL-	For	TTCTCAACCTCA GCACTGGTGACA	248	60
	Rev	GACTTTGCTGT TTGCTGTCAGGCT		

### qRT-PCR assay

2μg of the total RNA extracted from cells with RNeasy kit (Qiagen) was reversed transcribed with a Moloney murine leukemia virus reverse transcriptase (Promega Italia SrL, Milan, Italy) and subjected to qPCR with the following primers:

**Table:** 

Gene		5′-3′ Sequence	bp lenght	Tm
p53	For	AAGGAAATTT GCGTGTGGAGT	218/223	60
	Rev	AAAGCTGT TCCGTCCCAGTA		
Ccnb1	For	CACTTCCTTC GGAGAGCATC	240	60
	Rev	CAGGTGCTG CATAACTGGAA		
Ccnb2	For	CAGTTCCCAA ATCCGAGAAA	227	60
	Rev	TCTGAGACAAG CAGGAAGCA		
TopoIIa	For	TGGCAGAGGC AGAGAGAGTT	82	60
	Rev	TCAAAAAGCAC CATAGAGTTGC		
Cdc2	For	CTGGGGTCAG CTCGTTACTC	172	60
	Rev	ATTCCACTTC TGGCCACACT		
Mdm2 P1	For	TTTCGCAGCC AGGAGCACCGT	268	60
	Rev	GGGTCTCTT GTTCCG		
Mdm2 P2	For	CTTTTTCTC TGCTGATCCAG	105	64
	Rev	CAGGGTCTC TTGTTCCGAAGCTG		
Bax	For	GTCCGGGGAG CAGCCCAGAG	217	64
	Rev	CTCCATGTTAC TGTCCAGTTCGTCC		
Puma	For	ACGACCTCAAC GCACAGTACGAG	145	64
	Rev	TAATTGGGCTCC ATCTCGGG		
Cdkn1a	For	TGACCCTGAA GTGAGCACAG	183	60
	Rev	GGGAAAAGGC TCAACACTGA		
HPV18 E6	For	TAATAAGGTTG CCTGCGGTGC	161	60
	Rev	TTCTCTGCGTC GTTGGAGTC		
HPV18 E7	For	ACATTTACCA GCCCGACGAG	107	60
	Rev	GGTCGTCTGCT GAGCTTTCT		
Jun	For	AGCAGCAAAG AACTTTCCCG	148	60
	Rev	CGTCCTTCTT CTCTTGCGTG		
Jun B	For	TGGAACAGC CCTTCTACCAC	241	60
	Rev	GAAGAGGCG AGCTTGAGAGA		
Fos	For	TTACTACCAC TCACCCGCAG	109	60
	Rev	GACCGTGGGA ATGAAGTTGG		
E2F1	For	ATGTTTTCCTG TGCCCTGAG	155	60
	Rev	ATCTGTGGTG AGGGATGAGG		
cMyc	For	GAGGCTATTC TGCCCATTTG	120	60
	Rev	GCTGCTGGTT TTCCACTACC		
Elk1	For	CCACCTTCAC CATCCAGTCT	220	60
	Rev	TCTTCCGAT TTCAGGTTTGG		
Sp1	For	GAGAAAAC AGCCCAGATGCC	245	60
	Rev	GCGTTTCCCA CAGTATGACC		
Rpl19	For	ATGAGTATGC TCAGGCTTCAGA	376	60
	Rev	TCAGGTACAGG CTGTGATACA		
p63	For	ACGAAGATC CCCAGATGATG	141	60
	Rev	TGCTGTTGCCT GTACGTTTC		
p73	For	GCGTGGAAGGC AATAATCTC	185	60
	Rev	CAGGGTGAT GATGATGAGGA		

### Plasmids

The wt PGL3-HPV18-URR luciferase plasmid was a kind gift from Dr. Dan DiMaio (Department of Genetics, Yale University School of Medicine, New Haven, USA) [32]. Mutated URR plasmids were obtained by introducing single point mutations through SOE (Splice by Overlap Extension)-PCR. Briefly, mutant promoters have been created through two rounds of PCR: in the first round, we used two internal primers, containing the desired mutations, coupled with external primers, containing the desired restriction sites (XhoI, HindIII), in order to obtain two half fragments of the promoter; in the the second round, we used the two fragments, obtained by the first PCR round, as internal primers coupled with two external primers, in order to obtain the complete promoter embedding the mutations.

**Table:** 

Mutation	Fragment		Primer 5′-3′
Mut 1	Fragment 1	For	TGAACAAT**GT**G CGCGCC
		Rev HindIII	ATGCCAAGCTTA CTTAGATCGC
	Fragment 2	For XhoI	CCGGGCTC GAGATCCC
		Rev	GGCGCGCACA TTGTTCA
Mut 2	Fragment 1	For	ATTTTGAACA **CGGTT**CG CGCCTCTTTG GCGCA
		Rev HindIII	ATGCCAAGCTTAC TTAGATCGC
	Fragment 2	For XhoI	CCGGGCTCG AGATCCC
		Rev	AAGAGGCGCG **AACCG**TG TTCAAAATATG TAGGAGCAGTG
Mut 3	Fragment 1	For	ATTTTGA**CTG**ATTGG C**CG**GCCTCTTTGGCG CATATAAG
		Rev HindIII	ATGCCAAGCTT ACTTAGATCGC
	Fragment 2	For XhoI	CCGGGCTC GAGATCCC
		Rev	AAGAGGC**CG**GC CAAT**CAG**TCA AAATATGTAG GAGCAGTGCC
Mut 4	Fragment 1	For	TAATTGCAT A**CGGTT**CT TGTACAACTACTTTC
		Rev HindIII	ATGCCAAGCTTA CTTAGATCGC
	Fragment 2	For XhoI	CCGGGCTC GAGATCCC
		Rev	GTTGTACAA G**AACCG**TA TGCAATTAGCTTAAG

The obtained PCR fragments have been digested with XhoI and HindIII and cloned into the PGL3 luciferase plasmid. The mutated plasmids have been transformed in DH5α *Escherichia coli* and then sequenced.

### Transient transfections

The indicated plasmids were transfected into subconfluent Hela cells using Metafectene Pro (Biontex), according to the protocol provided by the manufacturer. Following 24 hours, cells were collected and lysed with transfection lysis buffer (1% TritonX 100, 25 mM GlyGly pH 7.8, 15 mM MgSO4, 4 mM EGTA pH 8). Proteins quantification was performed with Bradford reagent (Sigma Aldrich) and luciferase activity was measured [41].

### Analysis of gene expression profiles

Affymetrix gene expression profilings were performed in Hela cells before and after 72 hours from shCTR and shNF-YA infection (GeneChip® Human Genome U133 Plus 2.0). Raw data were retrieved from Geo Dataset GSE40215, published by Fleming et al. [25]. Biological replicates (triplicates) were grouped and processed: normalization (rma), quality controls, probe set filtering, finding differentially expressed probe sets and annotating those probe sets to gene symbols were performed using Bioconductor packages (Affy and Limma). Genes were defined as upregulated or downregulated when the fold change of shNF-YA versus shCTR profile was above 1.3 and FDR <0.05. KEGG analysis was performed using DAVID software with default settings.

### Statystical analysis

At least three independent biological experiments have been performed. The values represented in the histograms are the average of the biological replicates and the bars indicate the Standard Error of the Mean (SEM). Statistical significance was calculated using independent, two tailed Student t-test between the indicated samples.

## SUPPLEMENTARY FIGURES



## Abbreviations

HPV: Human Papilloma Virus
TF: Transcription Factor
LCR: Long Control Region
URR: Upstream Regulatory Region
