# Supplementary material for: NF-Y loss triggers p53 stabilization and apoptosis in HPV18-positive cells by affecting E6 transcription
Source: Oncotarget. 2016 Jun 13;7(29):45901–15. doi: 10.18632/oncotarget.9974 (PMC5216769; doi:10.18632/oncotarget.9974)
Supplement: Supplementary file 1 [file oncotarget-07-45901-s001.pdf]

NF-Y loss triggers p53 stabilization and apoptosis in HPV18-positive cells by affecting E6 transcription

SUPPLEMENTARY FIGURES

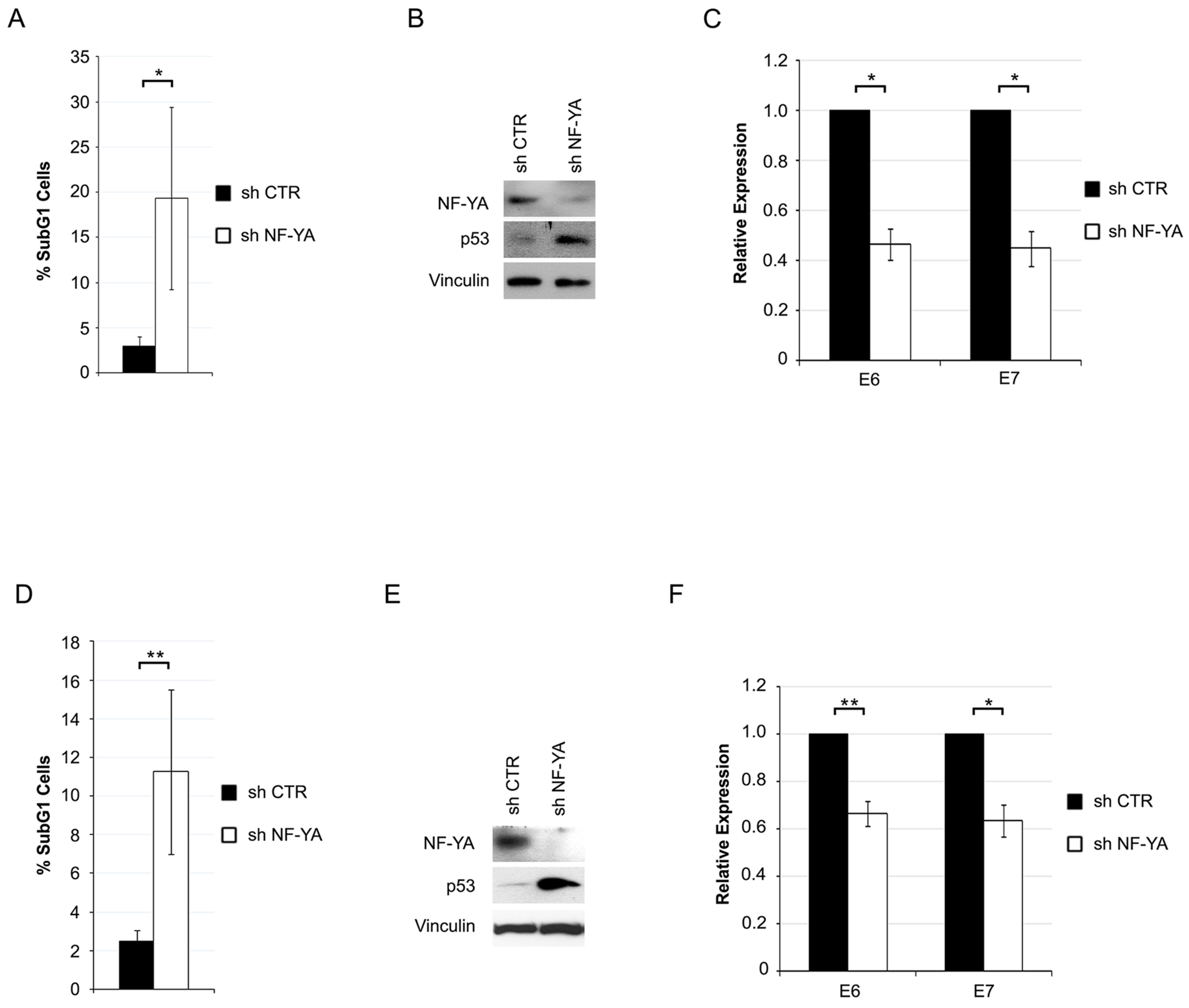

Supplementary Figure S1: A-C. NF-YA inactivation with a pool of NF-YA-targeting shRNAs. A. SubG1 events determined by Propidium Iodide staining of shCTR and shNF-YA cells upon 96h from lentiviral transduction. **B.** Western blot analysis of NF-YA and p53 expression in control and NF-YA-inactivated cells. **C.** qRT-PCRs of the indicated transcripts. Relative expression of transcripts has been calculated in shNF-YA versus shCTR, arbitrarily set at 1. **D-F. Effects of NF-YA inactivation in C4-I cells. D.** SubG1 events in shCTR and shNF-YA upon 96h from viral transduction. **E, F.** Protein and mRNA levels in NF-YA inactivated cells compared to control cells.

A

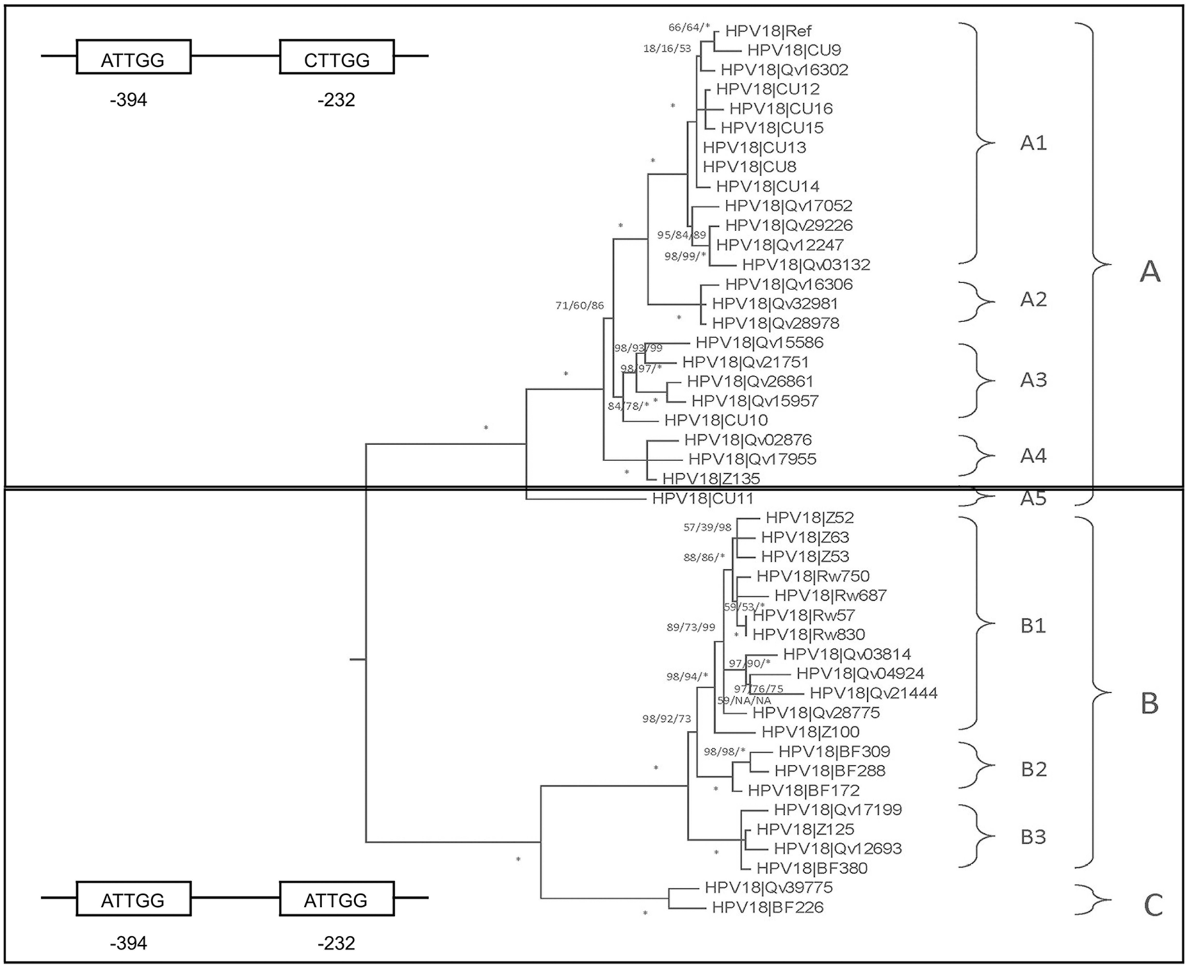

Supplementary Figure S2: Representation of the non canonical (upper box) or wt (lower box) status of the two identified NF-Y-motives within the LCR in the HPV18 phylogenetic tree (modified from Chen Z. et al. [9]).

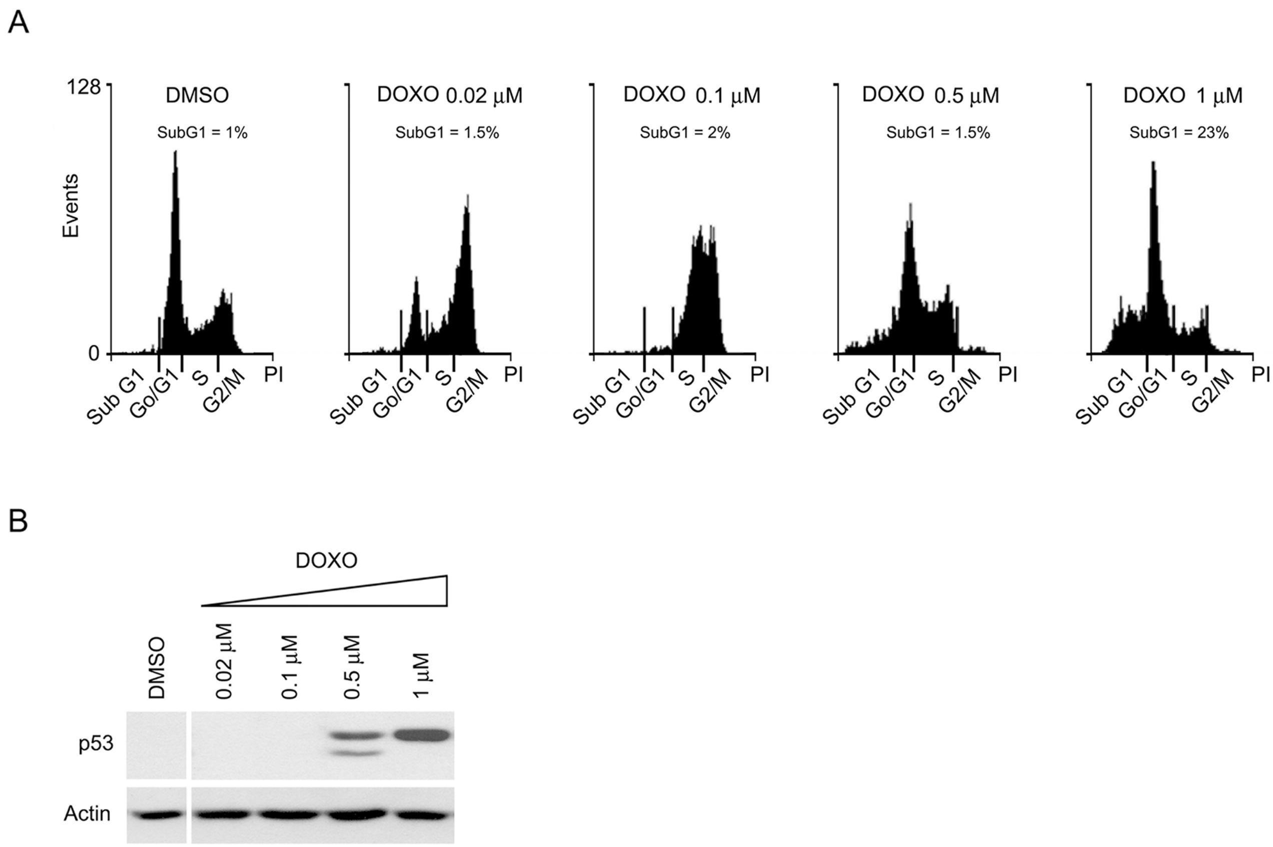

Supplementary Figure S3: A. DNA distribution analysis of Propidium Iodide-stained cells treated with increasing doses of Doxorubicin. The percentages of SubG1 events are indicated. **B.** p53 expression analysis in whole cell extract following Doxorubicin administration. Actin was used as loading control.

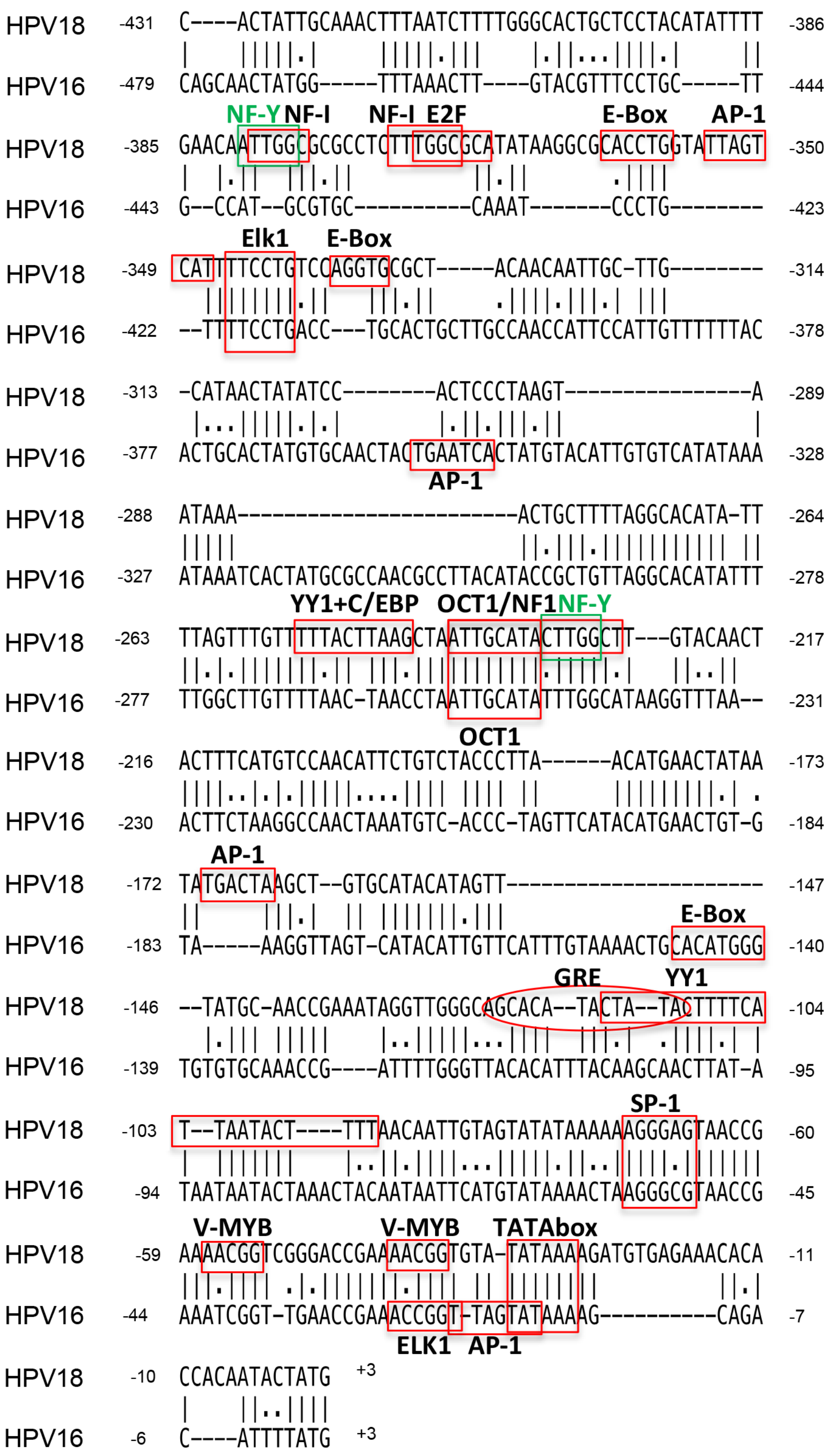

Supplementary Figure S4: Conservation of Transcription Factor Binding Sites (TFBS) within the URR of HPV18 and HPV16 integrated genomes. Sequence alignment of URR sequences from HPV18 (NC_001526) and HPV16 (AY262282) integrated genomes. The ATG sequence of E6 ORF has been indicated as +1. TFBS are highlighted in red boxes, NF-Y binding sites in green boxes.

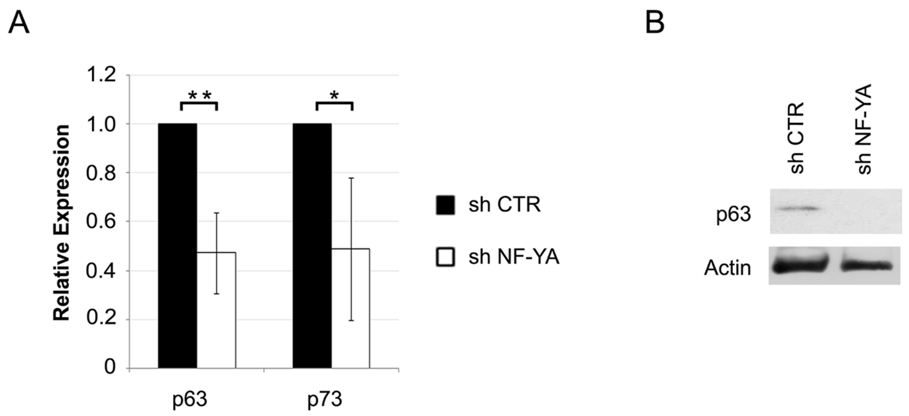

Supplementary Figure S5: Effect of NF-YA inactivation on p63 and p73 expression in Hela cells. A. mRNA expression levels of total p63 and p73 in control and NF-YA inactivated cells following 96h from viral infection. qRT-PCRs have been represented as expression levels of shNF-YA versus shCTR, arbitrarily set at 1. Statistical significance was calculated with independent t-test (* $p < 0.05$; ** $p < 0.01$). Error bars indicate SD. **B.** Western blot analysis of p63 protein levels in shCTR and shNF-YA cells.
